# Obturator Hernia: A Critical Appraisal of Treatment Strategies Based on 10 Cases and Current Literature: Towards a Pragmatic Surgical Algorithm

**DOI:** 10.7759/cureus.101888

**Published:** 2026-01-20

**Authors:** Yujo Kawashita, Eri Daicho, Noriko Ikeda, Masaki Tateishi, Takashi Ueda

**Affiliations:** 1 Surgery, Fukuoka Seisyukai Hospital, Fukuoka, JPN

**Keywords:** mesh repair, obstructed obturator hernia, open and laparoscopic surgery, small bowel resection, staged repair, transabdominal preperitoneal (tapp), treatment algorithm

## Abstract

Obturator hernia is a rare pelvic hernia that predominantly affects elderly, thin women. Despite advances in imaging and surgical techniques, the optimal surgical approach remains controversial. We retrospectively analyzed 10 patients who underwent emergency surgery for an obturator hernia at our institution between January 2010 and August 2024. All patients were female with a median age of 88.9 years (range: 84-97). Six patients (60%) required bowel resection. Seven underwent open repairs, and three underwent laparoscopic transabdominal preperitoneal (TAPP) repair. One patient with perforated peritonitis died postoperatively. During a median follow-up of 32 months (range: 18-51), one recurrence occurred at 34 months in a patient who had undergone suture repair without mesh reinforcement. Based on our experience and a comprehensive literature review encompassing three systematic reviews/meta-analyses and one nationwide registry study, we propose a treatment algorithm centered on three principles: TAPP as the default approach, bowel viability as the central decision point, and a contamination-based repair strategy. Two illustrative cases demonstrate the clinical application of this algorithm.

## Introduction

Obturator hernia accounts for 0.07-1% of all abdominal wall hernias [1,2]. The condition predominantly affects elderly, thin women because of the wider female pelvic outlet and age-related loss of preperitoneal fat in the obturator canal [3-6]. Among all abdominal wall hernias, the obturator hernia carries the highest mortality rate, ranging from 13% to 47% [1,7,8]. The classic Howship-Romberg sign is present in only 15-56% of cases, and this nonspecific presentation often leads to diagnostic delays [1,6,9]. A recent Danish nationwide registry study reported 14% 30-day mortality for emergency repairs [10]. With the aging population, the incidence has been gradually increasing [7]. Computed tomography (CT) has become the diagnostic gold standard, with preoperative detection rates exceeding 90% [11-15]. Recent systematic reviews and meta-analyses have shown advantages of laparoscopic repair over open surgery, with reduced morbidity (odds ratio 0.29) and mortality (odds ratio 0.84) [1-3]. However, the rarity of this condition and its emergency presentation preclude randomized controlled trials, and consensus on the optimal approach remains elusive. We aimed to analyze our institutional experience and propose a practical treatment algorithm based on current evidence.

## Materials and methods

This single-center retrospective case series included all consecutive patients who underwent emergency surgical repair for obturator hernia at Fukuoka Seishukai Hospital between January 2010 and August 2024. No patients were excluded. The study was conducted in accordance with the Strengthening the Reporting of Observational Studies in Epidemiology (STROBE) guidelines and approved by our institutional review board. We collected data on patient demographics, clinical presentation, time from symptom onset to surgery, operative findings, surgical approach, repair method, operative time, postoperative complications (graded by the Clavien-Dindo classification), length of hospital stay, and recurrence. All patients underwent preoperative CT. The choice of surgical approach was at the discretion of the operating surgeon based on clinical assessment of bowel viability and patient condition. Patients were followed at 1, 3, 6, and 12 months postoperatively and annually thereafter; data were supplemented by telephone interviews when clinic visits were not possible. Given the small sample size, we report only descriptive statistics.

We also performed a literature review focusing on systematic reviews, meta-analyses, and large cohort studies published between 2010 and 2024. We searched PubMed, MEDLINE, and the Cochrane Library using the following search terms: 'obturator hernia' AND ('systematic review' OR 'meta-analysis' OR 'cohort study' OR 'case series' OR 'treatment' OR 'laparoscopic' OR 'open repair' OR 'mesh repair'). We will also include studies reporting on surgical management, outcomes, and treatment strategies for obturator hernia. Reference lists of identified articles were also screened for additional relevant publications.

## Results

We identified 10 cases during the study period (Table 1).

**Table 1:** Summary of 10 Obturator Hernia Cases Onset-Op: time from symptom onset to surgery; Necrosis: bowel necrosis requiring resection (+) or not (-); LOS: length of hospital stay; FU: follow-up; Conv: conversion to open; TAPP: transabdominal preperitoneal repair; Lap→Ant: laparoscopic converted to anterior approach. *Recurrence at 34 months. §Conv: conversion to open (initial laparoscopic approach converted to open laparotomy due to intraoperative findings). †Initial suture repair followed by elective mesh repair at eight weeks. ‡Cases 8 and 9 correspond to Illustrative Cases B and A.

Case	Year	Age (y)	Onset-Op (h)	Bowel Resection	Approach	Op time (min)	Repair	Complication	LOS (d)	FU (mo)	Recur
1	2010	87	49	Yes	Open	70	Suture	Fatal	-	-	-
2	2011	94	39	Yes	Open	75	Suture	Pneumonia	41	25	No
3	2012	88	25	No	Open	103	Suture	None	14	46	Yes*
4	2012	85	16	No	Open	35	Suture	Pneumonia	24	49	No
5	2015	88	37	Yes	Open	60	Suture	None	4	36	No
6	2017	91	25	Yes	Conv§	63	Suture†	None	12	24	No
7	2019	84	14	No	TAPP	40	Mesh	None	11	51	No
8‡	2021	89	15	No	TAPP	55	Mesh	None	8	32	No
9‡	2021	86	41	Yes	Lap→Ant	116	Mesh Plug	None	19	18	No
10	2022	97	47	Yes	Open	45	Suture	Pneumonia	26	24	No

All patients were female, with a median age of 88.9 years (range: 84-97). The median time from symptom onset to surgery was 30.8 hours (range: 14-49). Six patients (60%) required bowel resection for intestinal necrosis. Seven patients underwent open repair via midline laparotomy, and three underwent laparoscopic transabdominal preperitoneal (TAPP) repair. One laparoscopic case was converted to an anterior approach for bowel resection (Case 9). Hernia defect closure consisted of suture repair in six cases and mesh repair in four (three flat mesh via TAPP, one plug mesh via anterior approach).

The mean operative time was 68.6 minutes (range, 35-116 minutes). Postoperative pneumonia occurred in three patients (30%), all managed with antibiotics alone without intensive care. One patient (Case 1) died on postoperative day one. She was an 87-year-old woman transferred from another hospital, already in septic shock from perforated peritonitis. Despite emergency laparotomy with bowel resection and hernia sac ligation, she did not recover. This case illustrates that mortality in obturator hernia is driven primarily by delayed diagnosis and advanced bowel necrosis rather than the surgical procedure itself. The mean hospital stay for survivors was 17.7 days (range: 4-41). Follow-up was complete in all nine survivors, with a median duration of 32 months (range: 18-51). One recurrence occurred at 34 months in a patient who had undergone suture repair without subsequent mesh reinforcement (Case 3).

Illustrative cases

We present two contrasting cases to illustrate our algorithm.

Case A: Bowel Resection With Mesh Repair (Case 9)

**Figure 1:**
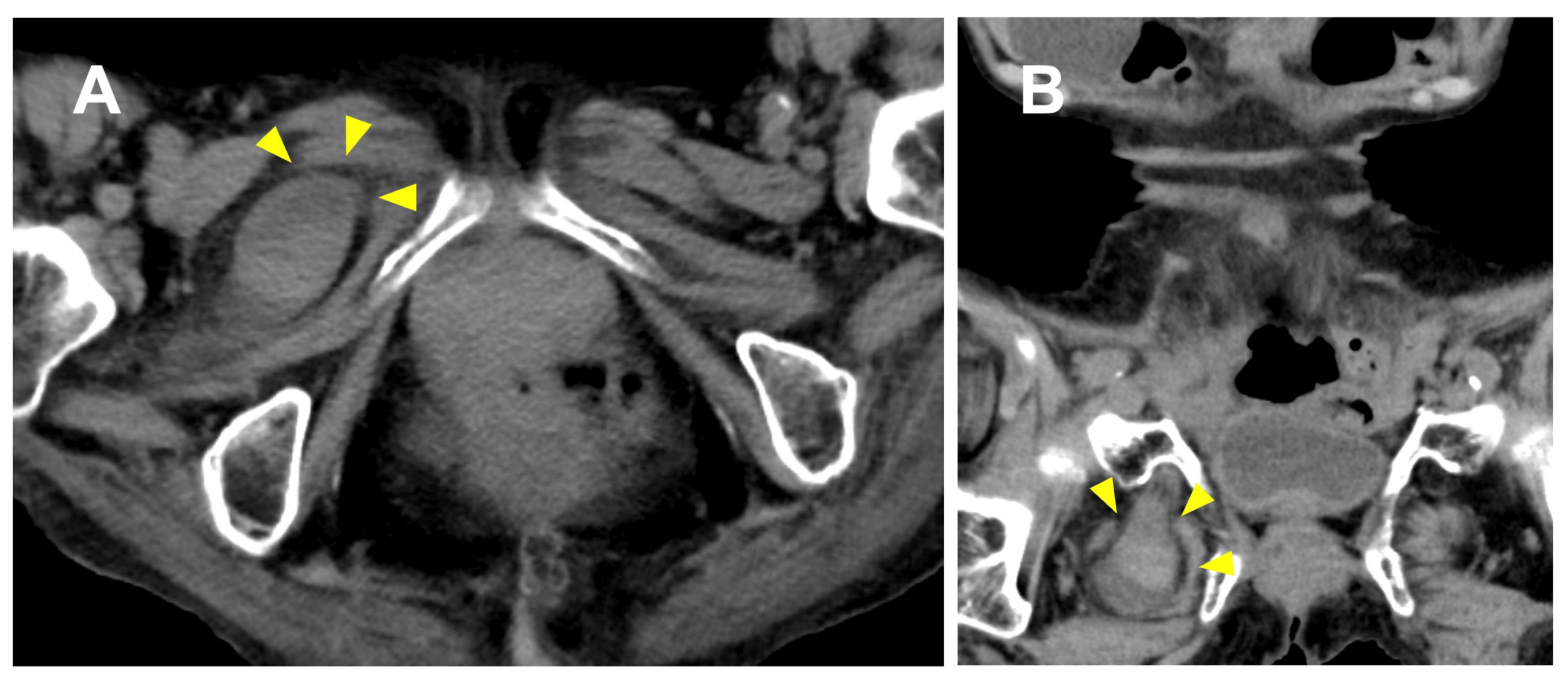
Case A: Preoperative CT. (A) Axial view showing right obturator hernia with incarcerated small bowel (arrowhead). (B) Coronal view.

An 86-year-old nursing home resident presented with vomiting and abdominal pain. Her history included hypertension, atrial fibrillation, and old cerebral infarction. Vital signs were stable, and the abdomen was soft with mild tenderness. Laboratory studies were unremarkable: white blood cell count 6,997/μL (reference: 3,300-8,600) and C-reactive protein 0.02 mg/dL (reference: <0.14). CT revealed a right obturator hernia with small bowel incarceration (Figure 1).

**Figure 2:**
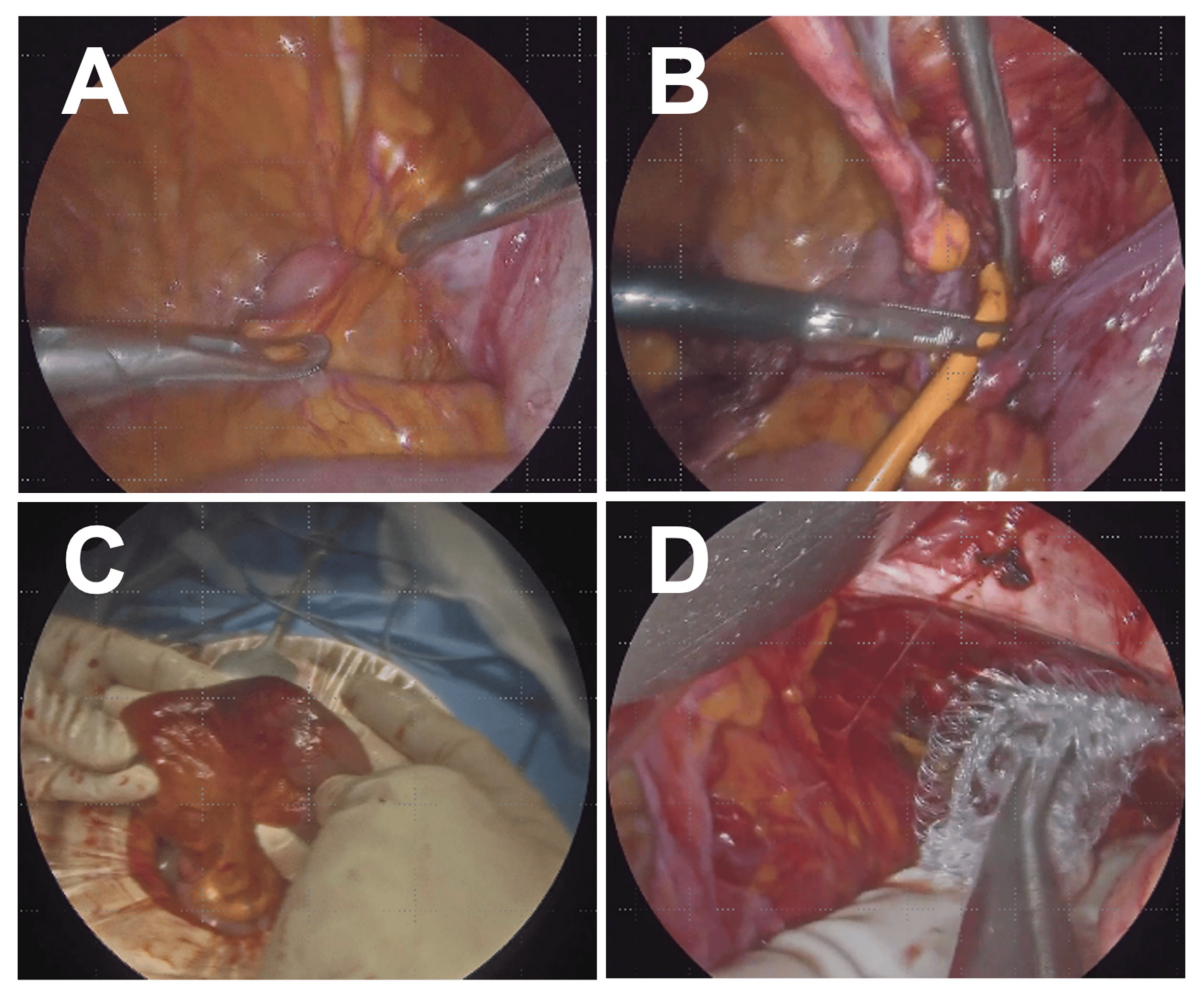
Case A: Operative Findings. (A) Laparoscopic view of ileal incarceration in the right obturator foramen. (B) Reduction using the water pressure technique. (C) Reduced bowel showing severe ischemic changes. (D) Mesh plug repair after conversion to anterior approach.

At laparoscopy, ileal incarceration was confirmed, and the bowel was reduced using the water pressure technique via the Nelaton catheter. However, the reduced segment showed dark purple discoloration without peristalsis or mesenteric pulsation. We converted to an anterior approach through an inguinal incision, performed a segmental ileal resection with functional end-to-end anastomosis, and repaired the defect with a mesh plug. There was no perforation, and spillage was minimal. According to the Centers for Disease Control and Prevention (CDC) wound classification system, this operative field was classified as Class II (clean-contaminated), defined as an operative wound in which the gastrointestinal tract is entered under controlled conditions without unusual contamination. The 2017 World Journal of Emergency Surgery (WSES) guidelines and supporting systematic reviews indicate that synthetic mesh placement is acceptable in clean-contaminated fields (CDC Class II) without significantly increased risk of surgical site infection [16-18]. Given the patient's overall clinical stability, the absence of frank peritonitis, and the controlled nature of the resection and anastomosis, we determined that primary mesh repair was appropriate and safe (Figure 2).

Operative time was 116 minutes. The patient was discharged on day 19 and remains recurrence-free at 18 months.

Case B: TAPP Repair With Bowel Preservation (Case 8)

**Figure 3:**
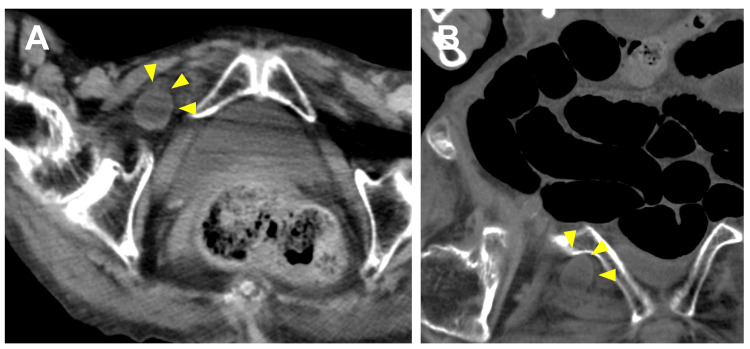
Case B: Preoperative CT. (A) Axial view showing right obturator hernia with incarcerated terminal ileum (arrowhead). (B) Coronal view.

An 89-year-old woman hospitalized for heart failure developed nausea and abdominal pain. CT showed a right obturator hernia with small bowel incarceration (Figure 3).

**Figure 4:**
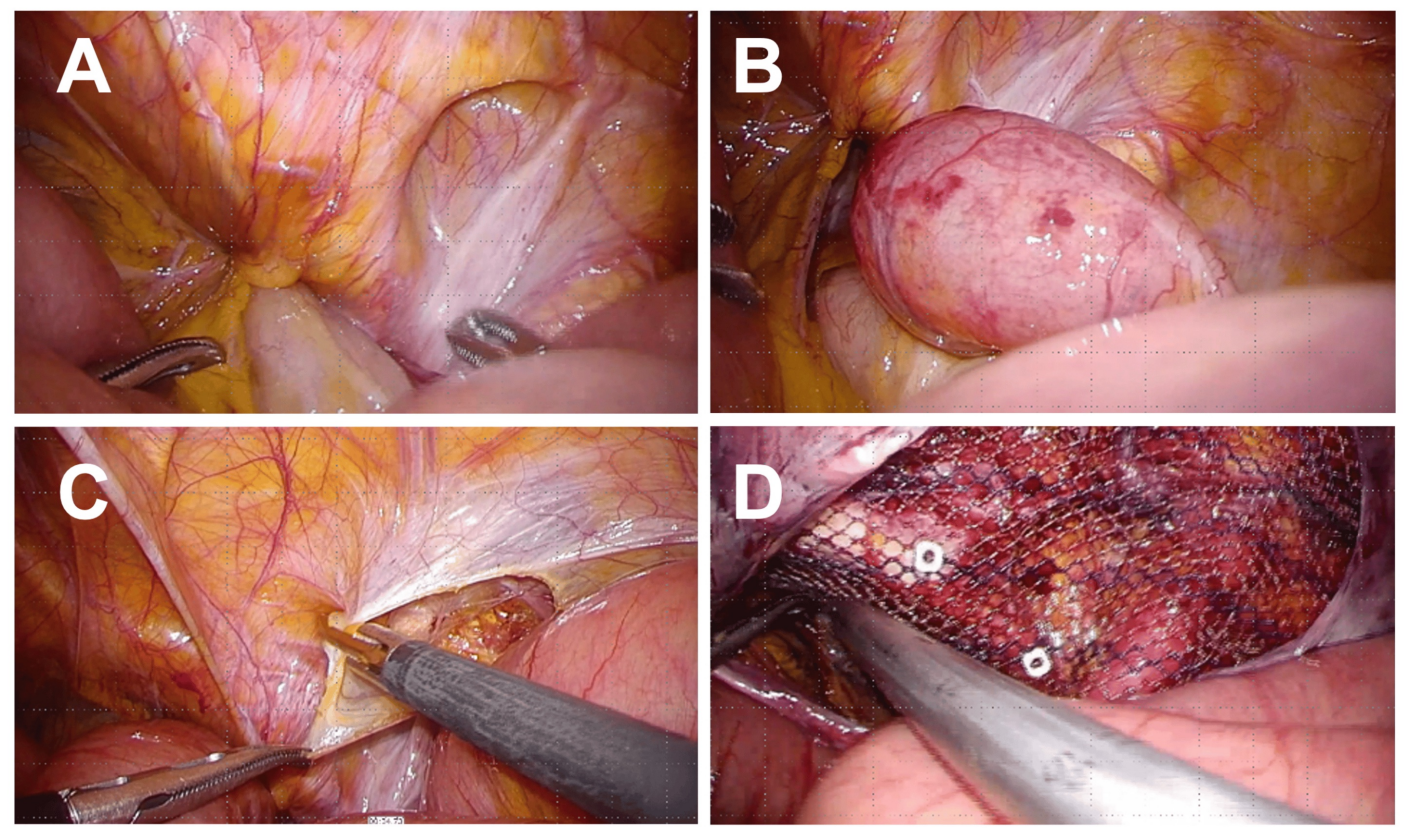
Case B: TAPP Repair. (A) Laparoscopic view of ileal incarceration. (B) After reduction, the bowel showed mild ischemia with prompt color recovery. (C) Preperitoneal dissection. (D) Flat mesh (15×10 cm) covering the obturator foramen and inguinal regions.

Laboratory studies showed mild inflammation: white blood cell count 13,792/μL and C-reactive protein 0.59 mg/dL. At laparoscopy, ileal incarceration was confirmed. Upon reduction, the bowel showed only mild ischemic changes with prompt color improvement and visible peristalsis; resection was unnecessary. We dissected the preperitoneal space and placed a 15×10 cm flat polypropylene mesh to cover the obturator foramen and inguinal regions, fixed with three tacks (Figure 4).

Operative time was 55 minutes. She was discharged on day eight and remains recurrence-free at 32 months.

## Discussion

Obturator hernia remains a formidable surgical emergency. Our series reflects the typical patient profile: elderly, thin women presenting with intestinal obstruction [1-7]. The 60% bowel resection rate and 10% mortality are consistent with published ranges of 15-50% and 13-47%, respectively [1,4,10,14,15].

Treatment has evolved considerably over the past decade. Three systematic reviews/meta-analyses now provide reasonable evidence for decision-making. Schizas et al. analyzed 725 patients from 47 studies and found that laparoscopic repair reduced morbidity (OR 0.29, 95% CI 0.09-0.88) and mortality (OR 0.84, 95% CI 0.27-2.60) compared with open surgery [1]. Burla et al. showed lower recurrence rates with mesh than suture repair [2]. Holm et al. reviewed 561 patients and reported no recurrences among 299 laparoscopic mesh repairs, versus 10% recurrence with open suture repair [3].

**Figure 5:**
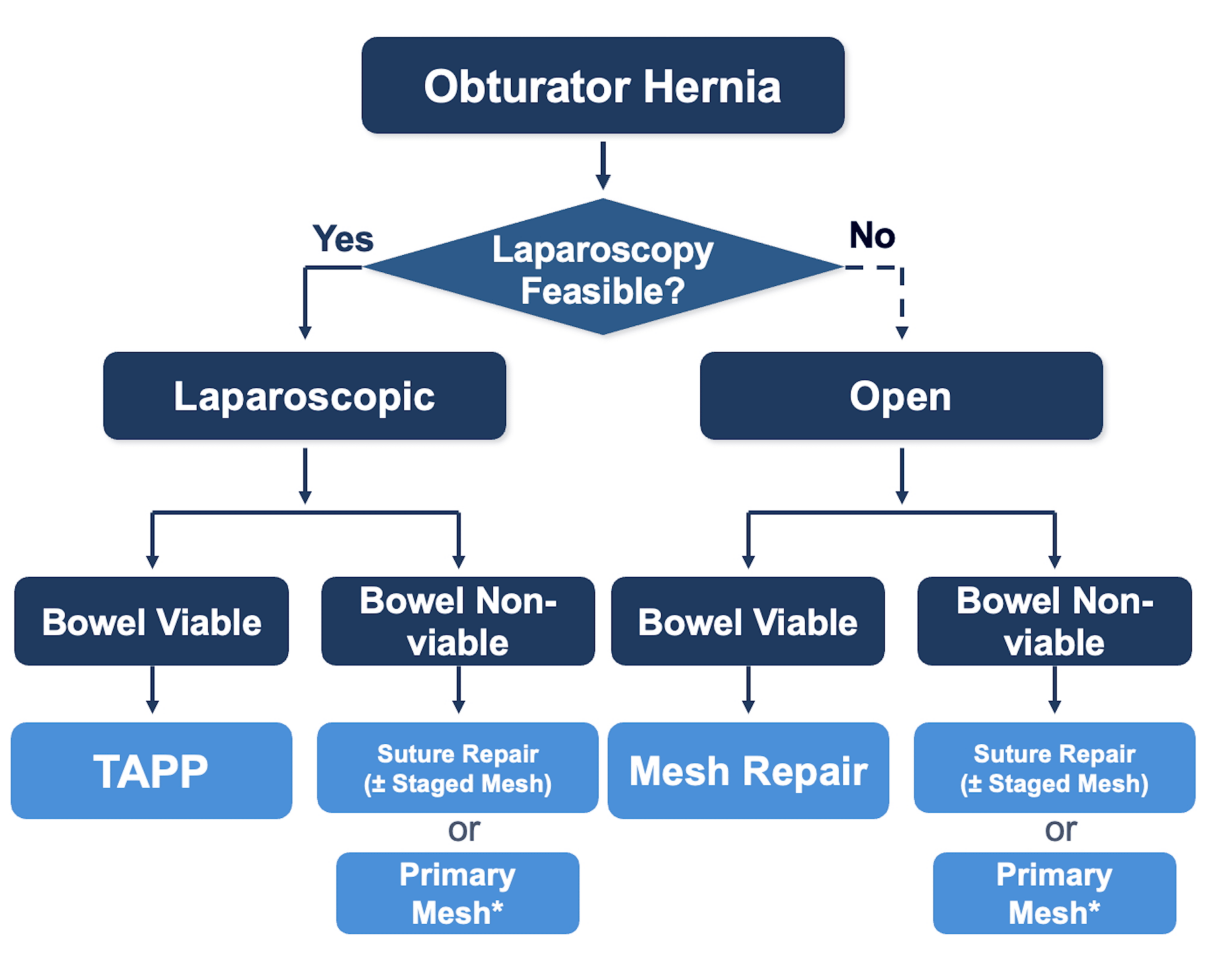
Treatment Algorithm. Laparoscopy is the first choice when feasible. Bowel viability is assessed by color, peristalsis, mesenteric pulsation, and bleeding. Repair strategy depends on contamination grade (CDC classification). Staged mesh may be omitted in patients with limited life expectancy. TAPP: transabdominal preperitoneal repair; CDC: Centers for Disease Control and Prevention. Figure created by the authors.

Based on this evidence and our experience, we believe the key question is not "laparoscopic versus open" but "does the patient need bowel resection?" (Figure 5).

When bowel viability is preserved, laparoscopic TAPP repair provides several practical advantages. When resection is required, the contamination status, not the approach itself, should guide mesh usage. This perspective has direct implications for intraoperative decision-making.

We generally favor laparoscopic TAPP as the initial approach when technically feasible. This preference is supported by convergent evidence from multiple systematic reviews, which report lower morbidity and mortality and, notably, no recurrences in nearly 300 laparoscopic mesh repairs [1-3]. TAPP also allows direct assessment of bowel viability and contralateral inspection before committing to a repair strategy.

The critical decision point is bowel viability. We assess this intraoperatively by examining tissue color (pink versus dark purple/black), peristalsis, mesenteric pulsation, and bleeding from cut edges. When two or more criteria suggest ischemia, we resect. If the bowel is viable, primary mesh repair carries a low infection risk, as in Case B. If resection is required, mesh placement depends on contamination. The 2017 WSES guidelines permit synthetic mesh in clean-contaminated fields (CDC wound class II) but advise against it in frankly contaminated or dirty fields (class III-IV) [16]. A systematic review found no increase in surgical site infection with mesh in clean-contaminated cases [17,18].

Our approach to mesh use is therefore graduated. With frank perforation and peritonitis (CDC class III-IV), we perform suture repair and plan staged mesh reinforcement at 6-8 weeks once infection has resolved. This strategy reflects concern that mesh infection in frail elderly patients can have serious consequences. When resection is performed for ischemic but non-perforated bowel with limited spillage (CDC class II), primary mesh may be considered. Case A exemplifies this scenario, in which controlled resection without perforation permitted safe mesh plug placement.

The fatal case (Case 1) was transferred already in septic shock from perforated peritonitis, a situation where the outcome is poor regardless of surgical technique. The single recurrence (Case 3) occurred at 34 months in a patient with suture repair who did not undergo planned mesh reinforcement, underscoring the importance of eventual definitive repair.

TAPP offers specific advantages for obturator hernia beyond the general benefits of minimally invasive surgery. The pelvic visualization is excellent, facilitating safe dissection around the obturator neurovascular bundle [19-22]. The contralateral side can be inspected, which matters because bilateral or metachronous obturator hernias occur in 6-25% of patients [23-25]. In Case 9, initial laparoscopy revealed non-viable bowel and guided the decision to convert, illustrating the value of a laparoscopic-first approach even when definitive repair is not completed laparoscopically.

We assessed bowel viability by conventional clinical signs. These are subjective, and emerging technologies such as indocyanine green fluorescence angiography may offer more objective assessment, though data specific to obturator hernia remain limited [26].

Regarding mesh coverage, the HerniaSurge guidelines recommend at least 15×10 cm with 2-3 cm midline crossover [27]. We tailor coverage based on the inguinal floor: if weakness is suspected, we cover the entire myopectineal orifice; if the floor appears solid, we limit coverage to the obturator foramen with at least 3 cm overlap. For TAPP, we use flat polypropylene mesh. Mesh plugs via the anterior approach are an option but require careful positioning [28,29].

This study has several inherent limitations. First, the sample size was small (n = 10), and the retrospective design limits statistical power and generalizability. Second, the study period spanned 14 years (2010-2024), during which surgical techniques, patient selection, and perioperative management evolved substantially, introducing clinical heterogeneity. Importantly, the choice of surgical approach was determined by the surgeon's judgment rather than randomization. Patients with more severe disease or compromised clinical status were more likely to undergo open surgery, resulting in unavoidable selection bias. As a consequence, meaningful direct comparison of outcomes between laparoscopic and open surgery within our cohort is not feasible. Therefore, conclusions regarding the relative advantages of laparoscopic repair are derived from external evidence, including published systematic reviews and meta-analyses, rather than from comparative analysis within our own series. Furthermore, the proposed algorithm represents an institutional approach and should be interpreted as a hypothesis-generating framework rather than a prospectively validated guideline.

Despite these limitations, several findings support the clinical relevance of the proposed algorithm. The demographic characteristics, surgical complexity, and outcomes of our cohort, including high resection rates and mortality, are consistent with those reported in the existing literature, suggesting that our series represents a typical clinical spectrum of this rare condition rather than an outlier population. In addition, the observed recurrence pattern reinforces the rationale for mesh reinforcement, aligning with prior reports.

Importantly, the algorithm is not derived solely from our limited case series but from a structured synthesis of accumulated institutional experience and evidence from published systematic reviews and meta-analyses. While the present study cannot establish the efficacy of the algorithm, it provides a literature-informed framework for decision-making in a clinical context where randomized controlled trials are impractical. As such, the algorithm should be viewed as a pragmatic proposal intended to guide clinical judgment and to serve as a foundation for future prospective validation.

## Conclusions

We propose an algorithm for obturator hernia centered on three principles: TAPP as the default approach, bowel viability as the central decision point, and a contamination-based repair strategy. Primary mesh is appropriate for clean and clean-contaminated fields; staged repair is preferred when there is frank contamination. Bowel viability should be assessed by tissue color, peristalsis, mesenteric pulsation, and bleeding. Mesh coverage should be tailored to the condition of the inguinal floor. Multicenter studies are needed to validate this approach.
